# Spermidine Prevents Heart Injury in Neonatal Rats Exposed to Intrauterine Hypoxia by Inhibiting Oxidative Stress and Mitochondrial Fragmentation

**DOI:** 10.1155/2019/5406468

**Published:** 2019-05-14

**Authors:** Nannan Chai, Hao Zhang, Lingxu Li, Xue Yu, Yan Liu, Yan Lin, Lina Wang, Jiamin Yan, Sazonova Elena Nikolaevna, Yajun Zhao

**Affiliations:** ^1^Department of Pathophysiology, Harbin Medical University, Harbin 150086, China; ^2^Department of Nursing, Medical School of Chifeng University, Chifeng 024000, China; ^3^Pathology Department, First Affiliated Hospital of Soochow University, Suzhou 215006, China; ^4^Department of Biochemistry, Harbin Medical University, Harbin 150086, China; ^5^Department of Pathophysiology, Qiqihar Medical University, Qiqihar, Heilongjiang 161006, China; ^6^Laboratory Center of Molecular Biology, Harbin Medical University, Harbin 150086, China; ^7^Department of Physiology, Far Eastern State Medical University, 680000, Russia

## Abstract

Intrauterine hypoxia (IUH) is a common intrauterine dysplasia that can cause programming of the offspring cardiovascular system. In this study, we hypothesized that placental treatment with spermidine (SPD) can prevent heart injury in neonatal offspring exposed to IUH. Pregnant rats were exposed to 21% O_2_ or 10% O_2_ (hypoxia) for 7 days prior to term or were exposed to hypoxia and intraperitoneally administered SPD or SPD+difluromethylornithine (DFMO) on gestational days 15-21. Seven-day-old offspring were then sacrificed to assess several parameters. Our results demonstrated that IUH led to decreased myocardial ornithine decarboxylase (ODC) and increased spermidine/spermine N^1^-acetyltransferase (SSAT) expression in the offspring. IUH also resulted in decreased offspring body weight, heart weight, cardiomyocyte proliferation, and antioxidant capacity and increased cardiomyocyte apoptosis and fibrosis. Furthermore, IUH caused mitochondrial structure abnormality, dysfunction, and decreased biogenesis and led to a fission/fusion imbalance in offspring hearts. In vitro, hypoxia induced mitochondrial ROS accumulation, decreased membrane potential, and increased fragmentation. Notably, all hypoxia-induced changes analyzed in this study were prevented by SPD. Thus, in utero SPD treatment is a potential strategy for preventing IUH-induced neonatal cardiac injury.

## 1. Introduction

Newborns with intrauterine growth restriction (IUGR) often experience adverse perinatal outcomes that present with an increased mortality risk. The clinical evidence of cardiovascular dysfunction in fetal and/or early neonatal life supports the notion of perinatal programming before the onset of significant cardiovascular disease (CVD) in adulthood [1, 2]. Intrauterine hypoxia (IUH) is the most common adverse intrauterine condition and occurs under various circumstances such as high-altitude pregnancy [3], preeclampsia, placental insufficiency, and any inflammatory condition during pregnancy caused by gestational diabetes or even maternal obesity [4]. A number of studies have reported that oxidative stress is the basis of fetal complications associated with low birth weight and developmental plasticity, excessive generation of reactive oxygen species (ROS), and/or a decrease in antioxidant defense, leading to indiscriminate damage to the developing fetus—all molecular mechanisms implicated in fetal programming of CVD [5]. Fetal hearts of pregnant rats suffering from prenatal hypoxia developed oxidative stress at the end of pregnancy, after which the offspring developed impaired peripheral artery relaxation and altered heart contractility in adulthood [6]. Mitochondria are the main organelles involved in the production and regulation of ROS. IUGR leads to increased oxidative stress in offspring rat hepatic mitochondria and impaired hepatic mitochondrial function [7]. A similar result was found in the pancreases of IUGR rat offspring [8]. More recently, a study showed that cardiac mitochondrial respiratory function was impaired in guinea pig offspring exposed to IUH [9]. A balance between mitochondrial fusion and fission is necessary to maintain normal mitochondrial morphology, number, and function in the heart [10]. Song et al. [11] revealed that cardiac-specific abrogation of either mitochondrial fusion (mitofusin 1 (Mfn1) and Mfn2) or fission (Drp1 ablation) in adult mouse hearts provoked lethal cardiac pathology. Papanicolaou et al. [12] further showed that transgenic mice deficient in cardiac-specific MFN1 and MFN2 starting from the late embryonic period displayed severe mitochondrial dysfunction on the 7th day after birth, developed cardiomyopathy, and all died within 14 days. However, much is yet to be discovered regarding the effects of prenatal hypoxia on neonatal cardiac mitochondrial dynamics and function.

Polyamines (PAs) include spermine (SP), spermidine (SPD), and their precursor putrescine (PU) and are present in all types of mammalian cells. Intracellular levels of PAs are maintained and tightly controlled by enzymes that catalyze rate-limiting steps of their biosynthesis by ornithine decarboxylase (ODC) and catabolism by spermidine/spermine-N1-acetyltransferase (SSAT) [13]. Polyamines are small polycations essential for all cellular life and are involved in gene expression [14], cell growth and differentiation [14], anti-inflammatory effects [15], antiapoptosis [16], protection against oxidative stress [17, 18], induction of autophagy [19], stabilization of cell and mitochondrial membranes [20], and embryonic development [21, 22]. Both SP and SPD can neutralize a wide spectrum of ROS including H_2_O_2_ [23], O_2_^·-^ [24], and HO^·^ [25, 26] as well as singlet oxygen [27]. We previously demonstrated that exogenous PAs reduce myocardial ischemia/reperfusion injury by inhibiting the production of ROS and opening of the mitochondrial permeability transition pores (mPTP) [28]. PAs can inhibit the H_2_O_2_-induced decrease in mitochondrial respiratory function in a concentration-dependent manner in cardiomyocytes [29]. It is noticeable that polyamine levels in the placental tissue of the sheep exposed to IUH were found decreased [21], while supplementation with exogenous PAs ameliorated embryonic dysplasia [22]. A recent study showed that L-arginine (Arg), a precursor of PA synthesis, is essential during pregnancy for growth and development of the conceptus and dietary supplementation of Arg during gestation was effective in improving embryonic survival and development of the conceptus in many species including humans, pigs, sheep, mice, and rats [30]. However, it is unclear whether administration of exogenous PAs to hypoxic mothers during pregnancy can reduce myocardial injury in newborn offspring.

Emerging evidence implicates that the important role of SPD in disease and health has received more attention recently [31, 32]. SPD, but not SP, is essential for early development and organogenesis [33], and SPD can significantly alleviate lipopolysaccharide-triggered inflammation in a zebrafish model [15]. Jamwal and Kumar revealed that rats administered with SPD at 5-10 mg/kg/day for 21 days intraperitoneally exerted neuroprotective effect in Huntington-like neuropathologies [34]. SPD (5 mg/kg ip) treatments in mice for 10 d led to a partial rescue of histological alterations in muscle defects [35]. Accordingly, 5 mg/kg/day SPD was selected to be given to the hypoxic mother during the late stage of pregnancy in order to study the protection of exogenous SPD on myocardial injury in newborn offspring suffered from maternal hypoxia.

## 2. Materials and Methods

### 2.1. Experimental Animals

All experiments were carried out following the National Institutes of Health guidelines, and all procedures were approved by the Local Ethics Review Committee of Harbin Medical University (China). Male and female Wistar rats (3 months old) were purchased from Harbin Medical University Experimental Center. Rats at a female : male ratio of 2 : 1 were randomly mated in one cage and tested for pregnancy via a vaginal smear obtained the following morning, which was examined for the presence of sperm, or through a copulatory plug found in the vagina, which signified day 0 of pregnancy. The pregnant rats were housed in rooms with controlled humidity (60%), controlled temperature (21°C), and a 12 : 12 h light-dark cycle.

### 2.2. Intrauterine Hypoxia Model

From day 15 to day 21 of pregnancy, the hypoxic rats (*n* = 9) were placed inside a plexiglass chamber with a maternal oxygen supply of 10% for 4 h every day. The percentage of oxygen in the plexiglass chamber was monitored by continuous infusion of a nitrogen gas and air mixture with an oxygen analyzer (Pro OX120; BioSpherix, New York, NY). We placed calcium chloride into the chamber to absorb excess carbon dioxide and water vapor for humidity control to avoid carbon dioxide retention and acidosis. In the hypoxic group, femoral artery intubation was performed in pregnant rats before exposure to hypoxia. The catheter consists of an artery catheter, a connecting tube, and a three-way valve. After successful intubation, the incision was covered with gauze. Then, the pregnant rats were exposed to hypoxia. During hypoxia, we adjusted the three-way valve to communicate with the syringe, blood was collected once every hour, and the collected blood was immediately dripped onto the blood gas testing chip (EG7+; Abbott, Lake Bluff, IL). The chip was inserted into the portable blood gas analyzer (i-STAT 200; Abbott); after which, the partial pressure of oxygen, blood oxygen saturation, and PH were measured. Arterial oxygen partial pressure of pregnant rats was maintained at 50-55 mmHg, and blood oxygen saturation was maintained at 80–85% [36–38]. This experimental procedure was carried out as a preexperiment to successfully establish our rat model of IUH. Prior to term (22 days), SPD or SPD+difluoromethylornithine (DFMO; an inhibitor of the key PA synthesis enzyme ODC) were administered intraperitoneally (ip) to the hypoxic rats for 7 days (gestational days 15–21). The control rats were maintained at room air conditions (21% O_2_) throughout pregnancy. The animals were randomly divided into four groups: (1) normoxic control group, control mother+0.9% saline (1 mL/kg/d; ip); (2) Hpx group, hypoxic mother+0.9% saline (1 mL/kg/d; ip); (3) Hpx-Spd group, hypoxic mother+SPD (5 mg/kg/d; ip); and (4) Hpx-SPD-DFMO group, hypoxic mother+SPD (5 mg/kg/d; ip)+DFMO (5 mg/kg/d; ip). After birth, newborn pups from the above four groups were reared with their mothers and housed under room air conditions; after which, they were sacrificed and their hearts were extracted on postnatal day 7 for subsequent experiments.

### 2.3. Histological Analysis

For histological analyses, ventricular tissue was fixed in 4% paraformaldehyde/phosphate-buffered saline (PBS) solution for 24-48 h. The tissue was subsequently dehydrated in a graded ethanol series, cleared in toluene, and embedded in paraffin. Five-micrometer paraffin sections were stained with hematoxylin-eosin (HE) and observed to assess overall cardiac morphology with an optical microscope (Eclipse E200; Nikon, Tokyo, Japan) [39]. Seven randomly chosen HE histological sections from each group of rats were analyzed by an expert pathologist.

### 2.4. Evaluation of Cell Proliferation by Immunohistochemical Analysis

For analysis of proliferation rates in neonatal hearts, immunohistochemical staining for MCM2 (minichromosome maintenance protein) was performed as previously described [40]. Briefly, after deparaffinization and rehydration of the tissues, inhibition of endogenous peroxidase was performed for 10 min with a solution of 0.3% hydrogen peroxide in PBS. Antigen retrieval was performed by heating for 5 min at 100°C in a citric acid buffer (0.01 mol/L in distilled water; pH 6.0). The tissues were incubated with 1% bovine serum albumin for 20 min and then incubated overnight with primary antibody against MCM2 (1 : 150, 105-1-AP, Proteintech, Wuhan, China). Between each subsequent incubation step, all slides were rinsed twice in PBS. The tissues were then incubated for 60 min with the HRP-labeled anti-rabbit IgG (1 : 200); after which, the slides were stained using a DAB Kit (AR1022, Boster, Wuhan, China) and counterstained with 0.1% hematoxylin for 5 s, followed by rinsing for 10 min with tap water. All tissues were analyzed by light microscopy after they were dehydrated, sealed with neutral gums, and dried.

### 2.5. TdT-Mediated dUTP Nick End Labeling (TUNEL) Assay

The TUNEL assay was used to determine the number of apoptotic cells in the 7-day-old neonatal rat hearts. Heart tissues were treated following our previously described procedure [28] and labeled with TUNEL (Upstate Cell Signaling Solutions, Lake Placid, NY) according to the manufacturer's instructions [39]. Cells were counted and scored from five randomly selected areas on each slide, followed by quantitative analysis using Image-Pro Plus (Media Cybernetics, Silver Spring, MD).

### 2.6. Quantification of Fibrosis

Cardiac tissue sections from neonatal rats were collected as previously described and deparaffinized, rehydrated, and stained with Masson's trichrome following standard protocols [41]. Cardiac fibrosis was assessed by Masson's trichrome staining. Two nonadjacent cross sections per heart were used, and fibrosis was analyzed as a percentage of the fibrotic area to the total left ventricular myocardial area using the ImageJ software, v1.52 (NIH, Bethesda, MD). Perivascular fibrosis was excluded from the analysis.

### 2.7. Transmission Electron Microscopy (TEM)

The cardiac apex tissues of neonatal rats in each group were dissected into approximately 1 × 1 × 1 mm pieces and randomly fixed in glutaraldehyde phosphoric acid buffer at 4°C for 2-4 h. The specimens were then washed, dehydrated, embedded in resin, stained with uranyl acetate and lead nitrate, sectioned into 50–70 nm sections, and finally visualized with a TEM (H600; Hitachi, Tokyo, Japan).

### 2.8. Mitochondrial Morphological Analysis and Area Measurement

Morphological analysis of mitochondria was performed as previously described [42]. Briefly, two left ventricle tissue pieces from each rat were randomly chosen. Images were acquired at a magnification of 10,000x when an area containing longitudinal myofilaments surrounded by a mitochondrial network was observed. Individual mitochondria and myofilaments were delineated.

### 2.9. Mitochondrial Isolation

Mitochondria were isolated at 4°C by differential centrifugation as our previously described using a mitochondria isolation kit (Beyotime Biotechnology, Shanghai, China) [29]. Briefly, the neonatal rat hearts were collected for weighing, followed by washing with PBS. The hearts were cut into small pieces in a petri dish on ice; after which, 10 volumes of precooled PBS were added in the samples and incubated in an ice bath for 3 min. The samples were then centrifuged at 600 ×*g* for 10-20 s to precipitate the tissue samples; the supernatant was discarded. After adding 8 volumes of precooled trypsin digest, the samples were incubated in an ice bath for 20 min and centrifuged again at 600 ×*g* for 10-20 s to precipitate the myocardial tissue; the supernatant was discarded. Eight volumes of mitochondrial separation reagent supplemented with phenylmethylsulfonyl fluoride (PMSF) before use were then added to the precipitate, homogenized 20–30 times in a homogenizer with an ice bath, and centrifuged for 5 min at 4°C. The supernatant was transferred to another centrifuge tube and centrifuged at 11,000 ×*g* for 10 min at 4°C; after which, the supernatant was carefully discarded, and the remaining precipitate contained the isolated mitochondria. The final cardiac mitochondrial pellet was then resuspended in isolation and homogenizing buffer, stored on ice, and used for experiments within 4 h. Protein concentrations were determined by the BCA Protein Assay (Beyotime Biotechnology).

### 2.10. Measurement of Mitochondrial Oxygen Consumption

Mitochondrial oxygen consumption was measured using a Clark-type oxygen electrode (Hansatech Instruments, Norfolk, UK) at 25°C in mitochondrial respiration buffer (125 mM KCl, 5 mM K_2_HPO_4_, 20 mM HEPES, 2.5 mM EGTA, and 1 mΜ MgCl_2_·6H_2_O; pH 7.2) as previously described [29]. Pyruvate (5 mM) was used as a substrate for complex I-containing mitochondria at a final concentration of 500 *μ*g protein/mL. ADP-stimulated oxygen consumption (state 3 respiration) was measured in the presence of 200 *μ*M ADP, and ADP-independent oxygen consumption (state 4 respiration) was monitored. The respiratory control ratio (RCR; state 3 divided by state 4) reflected oxygen consumption by phosphorylation (coupling).

### 2.11. Western Blot Analysis

To extract proteins from the 7-day-old neonatal rat heart tissues, frozen left ventricular cardiac tissues or isolated mitochondrial samples were homogenized in ice-cold RIPA lysis buffer (Beyotime Biotechnology) and 50 Lg/mL PMSF and then incubated on ice for 40 min. The homogenate was centrifuged at 10,000 ×*g* for 15 min at 4°C to remove cellular debris and isolate the total protein extract. Cardiomyocytes were harvested and lysed in the same RIPA buffer containing PMSF. Protein concentrations were determined using a Bradford assay kit (Beyotime Biotechnology) as previously described [43]. Briefly, equal amounts of protein from different experimental groups were loaded and separated via 10% SDS-PAGE; after which, the proteins were electrophoretically transferred to a 0.2 *μ*m pore polyvinylidene fluoride (PVDF) membrane (Merck Millipore, Burlington, MA). The membranes were blocked in TBS-T containing 5% (*w*/*v*) skim milk at 37°C for 1 h. Thereafter, the membranes were incubated overnight at 4°C with primary rabbit antibodies against GAPDH (1 : 2000; 10494-1-AP), VDAC (1 : 1000; 10866-1-AP), MFN2 (1 : 1000; 12186-1-AP), FIS1 (1 : 1000; 10956-1-AP), superoxide dismutase (SOD; 1 : 2000; 24127-1-AP), BAX (1 : 1000; 509599-2-Ig; all purchased from Proteintech, Chicago, IL), ODC (1 : 500; sc-33539), SSAT (1 : 1000; sc-67159), DRP1 (1 : 1000; sc-271583), BCL2 (1 : 2000; sc-7382; all purchased from Santa Cruz Biotechnology, Dallas, TX), and PGC-1*α* (1 : 1000; ab106814; Abcam, Cambridge, UK). Protein samples were fractionated using a PAGE Gel Rapid Preparation Kit (Beyotime Biotechnology) and transferred to PVDF membranes (Bio-Rad Laboratories, Hercules, CA). Samples were incubated overnight at 4°C with specific primary antibodies diluted in TBS-T. After washing, the samples were incubated with HRP-labeled goat anti-rabbit or HRP-labeled goat anti-mouse IgG secondary antibodies (Beyotime Biotechnology) at a dilution of 1 : 6000. Immunoreactive proteins were then developed using ultrasensitive ECL luminescent solution (Proteintech), quantified using a FluorChem Chemiluminescence Imaging System (ProteinSimple, San Jose, CA) via densitometry, and normalized to that of GAPDH. The final results were expressed as relative protein levels by normalizing the data to control values.

### 2.12. Quantitative Reverse Transcription PCR (qRT-PCR)

Cardiac tissue samples were homogenized in the TRIzol reagent (Invitrogen, Carlsbad, CA), and total RNA was isolated according to the manufacturer's instructions. mRNA was reverse transcribed with 2X SYBR Green qPCR Master Mix [44]. qPCR was performed on a LightCycler 96 Real-Time PCR System (Roche, Basel, Switzerland) according to the manufacturer's instructions. Gene-specific primers were designed using the Primer Express software (Thermo Fisher Scientific, Waltham, MA) and synthesized by Sangon Biotechnology (Shanghai, China) as follows, Mfn2, forward: 5′-CTC AGG AGC AGC GGG TTT ATT GTC T-3′ and reverse: 5′-TGT CGA GGG ACC AGC ATG TCT ATC T-3′; Fis1, forward: 5′-GTG CCT GGT TCG AAG CAA ATA C-3′ and reverse: 5′-CAT AAT CCC GCT GCT CCT CTT-3′; Drp1, forward: 5′-CGT AGT GGG AAC TCA GAG CA-3′ and reverse: 5′-TGG ACC AGC TGC AGA ATA AG-3′; and PGC-1*α*, forward: 5′-GTG CAG CCA AGA CTC TGT ATG G-3′ and reverse: 5′-GTC CAG GTC ATT CAC ATC AAG TTC-3′. All primers and PCR conditions were optimized to PCR efficiencies between 90% and 110% and a correlation coefficient of ≥0.990 using a cDNA dilution series. All samples were analyzed in triplicate. Relative mRNA levels were quantified using a standard curve and normalized to GAPDH levels. Relative gene expression was analyzed using the 2^-ΔΔCT^ method.

### 2.13. Hypoxic Cardiomyocyte Model

Neonatal rat hearts were enzymatically dissociated into a single cell suspension as previously reported [40]. Briefly, the hearts were rapidly excised and rinsed in PBS solution. The hearts were minced and incubated in 0.25% trypsin and 0.02% EDTA (Beyotime Biotechnology) at 37°C for 8 min. The obtained suspensions were then incubated for 2–3 min to precipitate undissociated tissue fragments. The supernatant was then centrifuged at 800 ×*g* for 10 min. The precipitate was transferred to DMEM supplemented with 10% fetal calf serum (Biolot, Saint Petersburg, Russia), 50 U/mL penicillin, and 50 *μ*g/mL streptomycin, followed by 1 h preincubation in a glass petri dish to eliminate nonmyocytic cells. The cells were plated at an initial density of 1 × 10^5^ cells/mL and cultured for 72 h in a CO_2_ incubator at 37°C and 5% СО_2_. The cells were then placed in a glass hypoxic chamber (BioSpherix OxyCycler C42; Redfield, NY), which was opened with an exhaust valve and filled with nitrogen for 8 min; no residual oxygen was found in the warehouse. The cultured neonatal rat cardiomyocytes (NRMCs) were then randomly divided into four groups: (1) control group (Con), cells cultured under normal incubation conditions; (2) hypoxia (Hpx) group, cells placed in a hypoxic chamber for 24 h and then cultured normally for 24 h; (3) Hpx-Spd group, cells placed in a hypoxic chamber and incubated with 10 *μ*mol/L SPD for 24 h; (4) and Hpx-Spd-DFMO group, cells placed in a hypoxic chamber and incubated with 10 *μ*mol/L SPD + 2 mmol/L DFMO for 24 h.

### 2.14. Mitochondrial Superoxide Production

MitoSOX Red is a mitochondrial superoxide indicator dye used to detect superoxide production in the mitochondria of living cells and determine the extent of oxidative stress [45]. The MitoSOX Red reagent is oxidized by superoxide in the mitochondria, leading to strong red fluorescence. NRCMs in each group were washed with PBS and incubated with 5 *μ*M MitoSOX Red according to the manufacturer's instructions. After a 10 min incubation period in the dark, the cells were washed again with PBS, followed by measuring MitoSOX Red fluorescence using excitation and emission wavelengths of 510 and 580 nm, respectively. Cardiomyocytes in each group were randomly imaged, and their total fluorescence was recorded and assayed.

### 2.15. Measuring ΔΨm

Isolated and cultured NRCMs were incubated with tetramethylrhodamine ethyl ester (JC-1; 1 *μ*g/mL) for 20 min in the dark in a cell incubator according to the manufacturer's instructions (Beyotime Biotechnology). NRCMs were washed twice with PBS and then immediately observed by fluorescence microscopy (BX51M; Olympus, Tokyo, Japan). Cardiomyocytes in each group were randomly imaged. Changes in ΔΨm were assessed by comparing the optical density ratios at 590–600 nm (red) to 527–534 nm (green) [46]. The ratio of intensity of the JC-1 aggregate (red) to that of the monomer (green) was calculated.

### 2.16. Mitochondrial Imaging

For mitochondrial staining, NRCMs were incubated in 200 nM MitoTracker® Green FM (dissolved in DMSO; Invitrogen) and preheated in culture medium at 37°C for 30 min as previously described [47, 48]. Briefly, the cells were washed and fixed with 4% paraformaldehyde for 10 min and then washed again and observed under a fluorescence microscope. An increase in the fragmentation pattern and mitochondrial number together with a decrease in mean mitochondrial volume was considered the criteria for mitochondrial fission. Within cells, two to three regions of interest (ROI) of an equal area were defined, and mitochondrial counts and volume were measured for each ROI. Each experiment was performed four times and 16–25 cells/condition were quantified.

### 2.17. Statistical Analysis

Data are expressed as the mean ± SEM. One-way analysis of variance was used for the statistical evaluation of differences among groups. Unpaired data were analyzed using Student's *t*-test where appropriate. All statistical analyses were performed using SPSS 13.1 (IBM, Armonk, NY) and GraphPad Prism 8 software (GraphPad Software Inc., La Jolla, CA). *P* values < 0.05 were considered statistically significant.

## 3. Results

### 3.1. PA Metabolism in the Neonatal Heart

**Figure 1:**
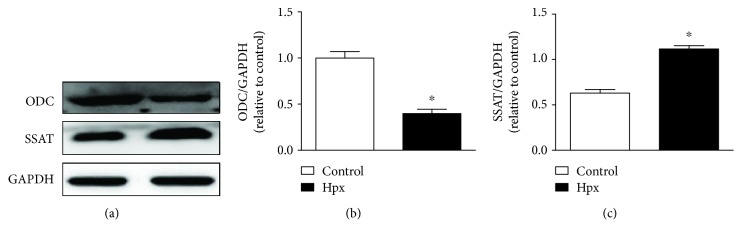
Polyamine metabolism in the neonatal rat hearts. Protein levels of ornithine decarboxylase (ODC) and spermidine/spermine acetyltransferase (SSAT) in cardiac tissue collected from neonatal offspring were determined by western blotting. Data are shown as the mean ± SEM; *n* = 4 per group. ^∗^ *P* < 0.05 versus control. Hpx: hypoxia.

We analyzed the effect of IUH on myocardial PA metabolism in the neonatal rats by determining the protein levels of the key PA synthesis enzyme ODC and the key PA catabolic enzyme SSAT in 7-day-old rats exposed to IUH (Figure 1). The results showed that ODC protein expression was decreased and SSAT protein expression was increased compared with those of the control group. These findings indicate that IUH altered myocardial PA metabolism in the neonatal offspring, leading to decreased PA synthesis and increased decomposition.

### 3.2. Offspring and Heart Characteristics

**Figure 2:**
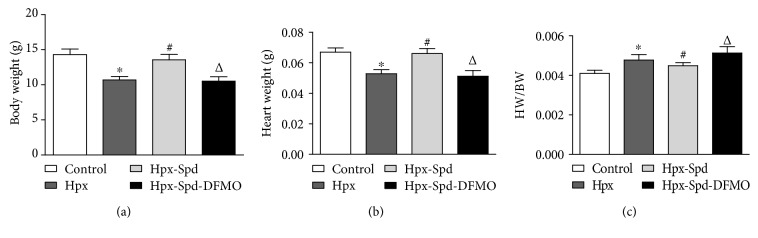
Evaluation of neonatal offspring and heart characteristics. (a) Body weight (BW), (b) heart weight (HW), and (c) BW/HW ratio of neonatal rats. Data are shown as the mean ± SEM; *n* = 8 per group. ^∗^ *P* < 0.05 versus control, ^#^ *P* < 0.05 versus the Hpx group, and ^△^ *P* < 0.05 versus the Hpx-Spd group. Hpx: hypoxia; Hpx-Spd: hypoxia and SPD treatment; Hpx-Spd-DFMO: hypoxia and SPD+DFMO treatment.

Body weight (BW) and heart weight (HW) of 7-day-old rats was measured and the HW to BW ratio (HW/BW) was calculated (Figure 2(c)). IUH led to decreased BW and HW (*P* < 0.05), while HW/BW increased in the offspring heart (*P* < 0.05). Exogenous SPD administration increased BW and HW of neonatal rats (*P* < 0.05) and significantly decreased HW/BW compared with that of the hypoxia group (*P* < 0.05). Meanwhile, the PA synthesis inhibitor DFMO abolished the effects induced by SPD (*P* < 0.05). These results demonstrate that chronic hypoxia during pregnancy resulted in offspring growth retardation and that SPD treatment during pregnancy inhibited changes caused by IUH, indicating that PAs can prevent the growth retardation induced by IUH.

### 3.3. Effects of SPD on Myocardial Morphological Structure, Cell Proliferation, Apoptosis, and Fibrosis in Offspring Exposed to IUH

**Figure 3:**
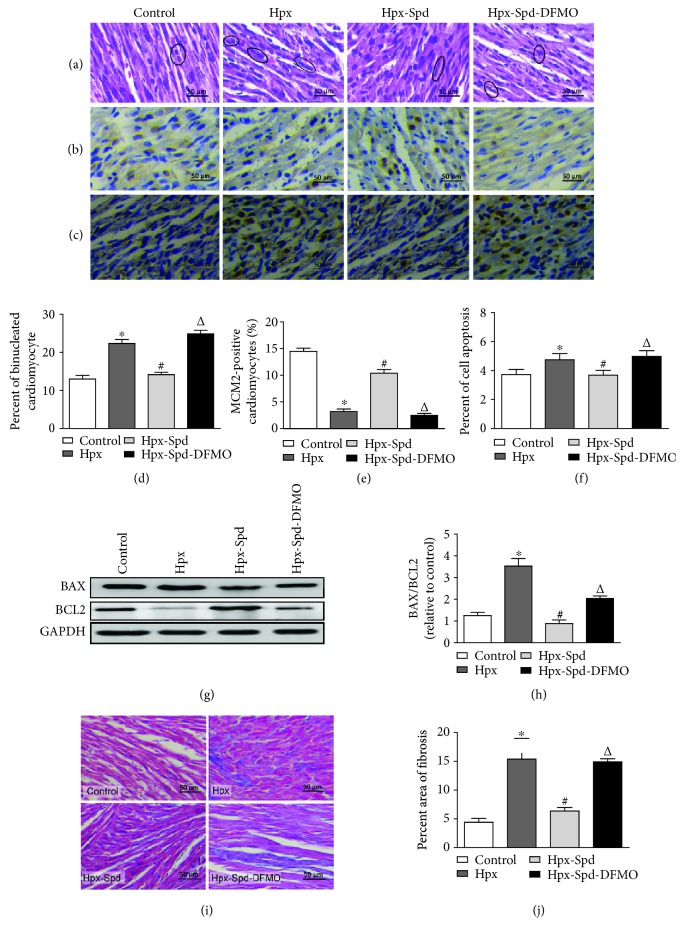
Observation of myocardial morphological structure, cell proliferation, apoptosis, and fibrosis in offspring rats. (a) Representative left ventricle sections stained by HE in the normal (control), hypoxia (Hpx), SPD-treated (Hpx-Spd), and SPD+polyamine synthesis inhibitor- (Hpx-Spd-DMFO-) treated groups. (b) Representative immunohistochemical staining for protein expression and localization of MCM2-positive cardiomyocytes. (c) Brown-stained nuclei indicate TUNEL-positive cells. (d) Evaluation of the percentage of binucleated cardiomyocytes (*n* = 6). (e) Evaluation of the percentage of MCM2-positive cells (*n* = 10). (f) The percentage of TUNEL-positive nuclei in different groups (*n* = 8). (g) BAX and BCL2 protein expression detected by western blotting. (h) Quantification of the BAX and BCL2 protein level ratio (*n* = 4). (i) Representative Masson's trichrome staining in ventricle sections in each group. (j) Evaluation of interstitial fibrotic areas in ventricle sections in each group (*n* = 8). Data are shown as the mean ± SEM. ^∗^ *P* < 0.05 versus control, ^#^ *P* < 0.05 versus the Hpx group, and ^△^ *P* < 0.05 versus the Hpx-Spd group.

Myocardial morphological structure, cell proliferation, apoptosis, and myocardial fibrosis were analyzed in the rat hearts of 7-day-old offspring. Compared with normal hearts, neonatal offspring hearts exposed to IUH showed loosely arranged cardiac muscle fibers and increased interstitial distances as evaluated by HE staining. However, hearts in the IUH group treated with SPD maintained good myocardial histological structures compared with those of control hearts (Figure 3(a)). We also examined the number of binucleated cardiomyocytes and expression of the nuclear proliferation protein MCM2 as they reflect the degree of cardiomyocyte maturation and differentiation. Analysis of the HE-stained samples showed a higher proportion of binucleated cardiomyocytes in rat hearts exposed to IUH compared with control hearts (*P* < 0.05). Meanwhile, the proportion of binucleated cardiomyocytes was significantly reduced in the hearts of offspring with SPD-treated mothers compared with heart suffered from IUH (*P* < 0.05); DFMO attenuated the effects of SPD (*P* < 0.05; Figures 3(a) and 3(d)). MCM2 expression in the IUH group was also significantly lower than that in the control group (*P* < 0.05) and was strongly expressed in the SPD group; DFMO again abolished the effect of SPD on MCM2 expression (Figures 3(b) and 3(e)). These results indicate that the PA SPD can promote myocardial proliferation in neonatal offspring exposed to IUH.

We further counted TUNEL-positive nuclei to determine the cell apoptosis ratio (Figures 3(c) and 3(f)) and found that the number of TUNEL-positive cardiomyocytes was substantially higher in neonatal rat hearts exposed to IUH than in control rat hearts (*P* < 0.05). In contrast, the ratio of TUNEL-positive cells in SPD-treated rats (*P* < 0.05) was lower than that in untreated hypoxic rats. As mitochondrial damage can induce the mitochondrial pathway of apoptosis, we analyzed the expression levels of proapoptotic protein BAX and antiapoptotic protein BCL2 in neonatal hearts by immunoblotting (Figures 3(g) and 3(h)) and found that the BAX/BCL2 protein expression ratio was significantly higher in the myocardium of the IUH group than of the control group (*P* < 0.05). However, in the myocardium of offspring exposed to IUH and administered with SPD, the BAX/BCL2 ratio was decreased compared with the IUH group (*P* < 0.05); expectedly, DFMO abolished the effects of SPD (*P* < 0.05). These results indicate that the PA SPD can inhibit cardiomyocyte apoptosis in the myocardium of neonatal offspring exposed to IUH.

Next, we evaluated myocardial fibrosis using a collagen area assay (Figures 3(i) and 3(j)) and observed increased myocardial collagen deposition in neonatal rat hearts exposed to IUH, which was higher than that of the control group (*P* < 0.05). Conversely, the area of fibrosis was decreased to a greater extent after SPD treatment compared with the IUH group (*P* < 0.05). Moreover, compared with the SPD treatment group, the Hpx-Spd-DFMO group showed a significantly enhanced fibrotic area (*P* < 0.05). Overall, our results suggest that SPD ameliorates the decreased proliferation as well as the increased apoptosis and collagen deposition in the offspring heart exposed to IUH.

### 3.4. Effects of SPD on Mitochondrial Structure and Function as well as SOD/ROS Production and ΔΨm in Offspring and Primary Cardiomyocytes Exposed to Hypoxia

**Figure 4:**
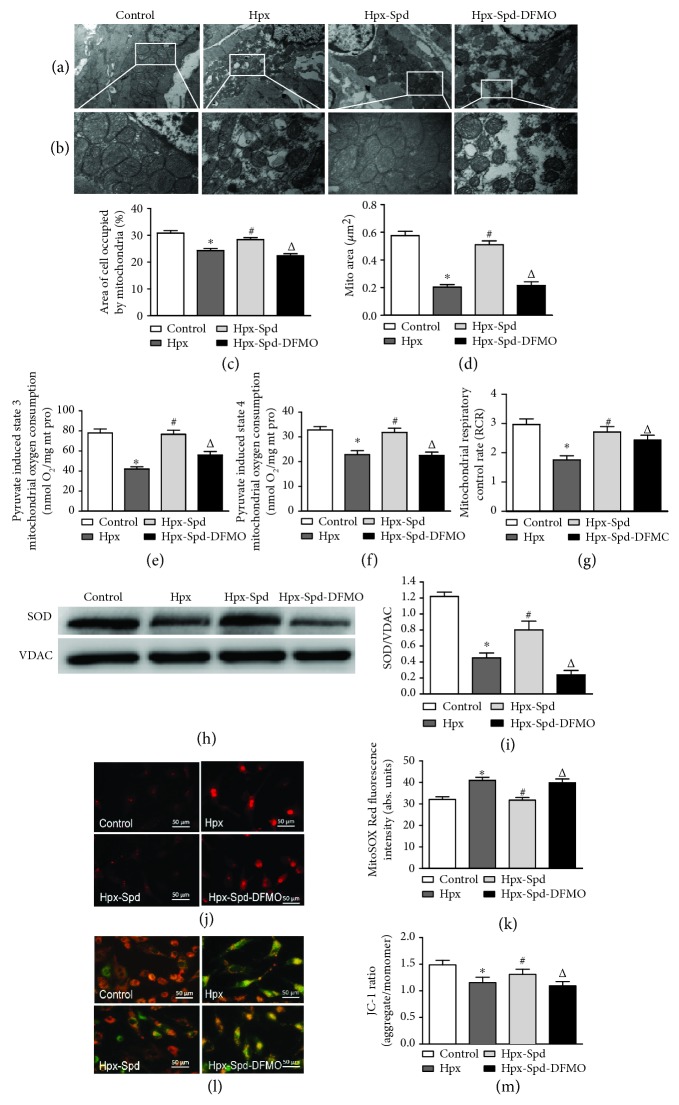
Effects of SPD on mitochondrial quantity and quality, mitochondrial function, and MnSOD expression in the myocardium of neonatal offspring and on mitochondrial ROS production and membrane potential in primary cardiomyocytes (NRCMs). (a) TEM of myocardial ultrastructure of normal (control), hypoxia (Hpx), SPD-treated (Hpx-Spd), and SPD+polyamine synthesis inhibitor- (Hpx-Spd-DMFO-) treated groups (magnification, 10,000x). (b) Representative TEM images showing ultrastructural changes in the myocardial mitochondria of each group (magnification, 30,000x). (c, d) Quantification of the area of cells occupied by mitochondria (%) and the quantitative mitochondrial area (*n* = 10). (e–g) Mitochondrial function was evaluated based on mitochondrial state 3 (e) and state 4 (f) oxygen consumption and the respiratory control rate (RCR) (g) in cardiac mitochondria isolated from 7-day-old offspring hearts (*n* = 10). (h) The expression ratio of SOD proteins in isolated mitochondria detected by western blotting. (i) Quantification of the protein levels (*n* = 4). (j) Detection of superoxide production in myocardial mitochondria of NRCMs by the MitoSOX Red fluorescent probe. (k) Statistical quantification of the average fluorescence intensity of MitoSOX Red (*n* = 6). (l) Detection of mitochondrial membrane potential (ΔΨm) of NRCMs by the JC-1 fluorescent probe. (m) Statistical analysis of the ratio of red to green fluorescence (*n* = 6). Data are shown as the mean ± SEM. ^∗^ *P* < 0.05 versus control, ^#^ *P* < 0.05 versus the Hpx group, and ^△^ *P* < 0.05 versus the Hpx-Spd group.

Changes in the myocardium and myocardial mitochondrial ultrastructure in neonatal rats were analyzed by TEM (Figures 4(a) and 4(b)). Mitochondrial content (% of the mitochondrial area compared with the whole-cell area) and mitochondrial area were quantified using ImageJ (Figures 4(c) and 4(d)). In normal rats, well-developed myocardial mitochondria with preserved membranes and cristae were observed; in contrast, decreased mitochondrial matrix density, disorganized mitochondrial cristae, and swollen, small, and irregular mitochondria were observed in some cardiomyocytes of hypoxic rats. However, in offspring hearts exposed to IUH and SPD treatment, the mitochondria were more tightly packed between the myofibrils and exhibited intact outer and inner membranes with distinct cristae. Compared with those of the IUH group, the proportion of mitochondria in myocardial cells and the mitochondrial area increased following SPD treatment; DFMO inhibited these SPD-induced effects.

We next measured mitochondrial respiratory function, including state 3 and 4 respiratory rates and the RCR (Figures 4(e)–4(g)), using pyruvate/malate as substrates. We found that state 3 and 4 respiratory rates as well as the RCR were significantly decreased in the IUH group compared with the control group (*P* < 0.05). Interestingly, state 3 and 4 respiratory rates and the RCR were restored by SPD treatment (*P* < 0.05); conversely, these SPD effects were significantly inhibited in the DFMO-treated group. These findings suggest that SPD can inhibit the decrease in mitochondrial respiratory function of neonatal rat hearts exposed to IUH.

We further determined the expression levels of the antioxidative enzyme SOD in the offspring myocardium (Figures 4(h) and 4(i)) and found that SOD protein expression in the myocardium of the IUH group was significantly decreased compared with that of the control group (*P* < 0.05). In the presence of SPD treatment, however, SOD levels in the IUH offspring myocardium were found increased (*P* < 0.05). DFMO once again abolished the effects of SPD (*P* < 0.05). The results indicate that in utero PA treatment can reverse the decrease in offspring myocardial antioxidants induced by IUH exposure.

Next, we examined mitochondrial ROS levels in vitro using MitoSOX Red (Figures 4(j) and 4(k)) and found that ROS production increased after cardiomyocytes were exposed to hypoxia for 24 h; conversely, SPD inhibited the hypoxia-induced ROS production, which was relieved when cardiomyocytes were also treated with DFMO. Furthermore, we measured ΔΨm in neonatal rat cardiomyocytes to evaluate the effects of SPD on ΔΨm depolarization in hypoxic cardiomyocytes (Figures 4(l) and 4(m)). The JC-1 fluorescent probe was used to label cardiomyocytes and monitor changes in ΔΨm. Red fluorescence indicated that the mitochondrial membrane was intact, whereas green fluorescence indicated that it was destroyed and ΔΨm was decreased; the red and green fluorescence ratios represent ΔΨm. We found that hypoxia exposure decreased ΔΨm, whereas SPD inhibited the hypoxia-induced decrease in ΔΨm.

### 3.5. Effects of SPD on Mitochondrial Biogenesis and Fission/Fusion Dynamics in Offspring and Primary Cardiomyocytes Exposed to Hypoxia

**Figure 5:**
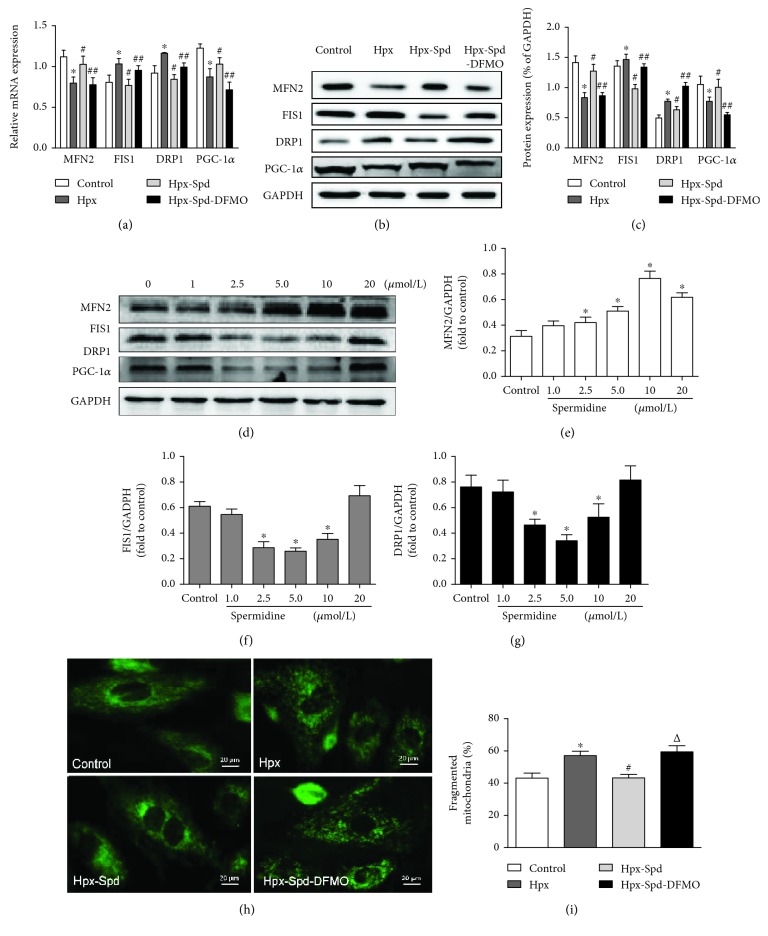
Effects of SPD on mitochondrial biogenesis and fission/fusion dynamics in the offspring myocardium and NRCMs exposed to hypoxia. (a) mRNA expression levels of myocardial MFN2, FIS1, DRP1, and PGC-1*α* were determined by qRT-PCR; GAPDH was used as an internal control (*n* = 6). (b) Protein levels of MFN2, FIS1, DRP1, and PGC-1*α* in the offspring myocardium collected from the control, Hpx, Hpx-Spd, and Hpx-Spd-DFMO groups were assessed by western blotting. (c) Quantification of MFN2, FIS1, DRP1, and PGC-1*α* protein levels normalized to GAPDH in each group (*n* = 4). Data are shown as the mean ± SEM. ^∗^ *P* < 0.05 versus control, ^#^ *P* < 0.05 versus the Hpx group, and ^△^ *P* < 0.05 versus the Hpx-Spd group. (d) Western blotting showed a dose-dependent effect of SPD (0, 2.5, 5, 10, and 20 *μ*mol) on the protein levels of MFN2, FIS1, and DRP1 in NRCMs after exposure to hypoxia. Quantitative analysis of (e) MFN2, (f) FIS1, and (g) DRP1 protein levels normalized to that of GAPDH. Data are shown as the mean ± SEM. ^∗^ *P* < 0.05 versus the control group (0 *μ*mol/L SPD treatment; *n* = 6). (h) Detection of fused and fragmented mitochondria in NRCMs by MitoTracker Green staining (magnification, 400x). (i) The percentage of fragmented mitochondria. *n* = 6 for each group. Data are shown as the mean ± SEM. ^∗^ *P* < 0.05 versus the control group, ^#^ *P* < 0.05 versus the Hpx group, and ^△^ *P* < 0.05 versus the Hpx-Spd group.

Mitochondrial morphology is regulated by a family of mitochondrial fusion and fission proteins; therefore, we investigated expression changes in the mitochondrial fusion protein MFN2, the fission proteins FIS1 and DRP1, and the mitochondrial biogenesis key regulatory protein PGC-1*α* at the mRNA and protein levels (Figures 5(a)–5(c)). We found that the mRNA levels of MFN2 and PGC-1*α* were significantly decreased, whereas those of FIS1 and DRP1 were increased in the hypoxia group compared with the control group (*P* < 0.05). Administration of SPD increased MFN2 and PGC-1*α* mRNA levels and decreased FIS1 and DRP1 levels compared with that of the hypoxia group (*P* < 0.05); conversely, DMFO treatment abolished the effects induced by SPD (*P* < 0.05; Figure 5(a)). Similarly, the changes in MFN2, FIS1, DRP1, and PGC-1*α* levels at the protein level were consistent with the trend observed for mRNA (Figures 5(b) and 5(c)).

We further examined the effects of different concentrations of SPD on the expression of mitochondrial fusion and fission proteins in primary cultured NRCMs. We found that SPD increased Mfn2 and decreased Fis1 and Drp1 expression in a dose-dependent manner (Figure 5(d)). The optimal concentration of SPD for promoting mitochondrial fusion and reducing fission was 10 *μ*mol and 5 *μ*mol, respectively (Figures 5(e)–5(g)). Therefore, 5 *μ*mol SPD was used to examine the effect of SPD on mitochondrial fusion and fission in hypoxic NRMCs using the MitoTracker® Green fluorescence probe. The results demonstrated that hypoxia disrupted the microtubule networks, whereas SPD treatment significantly inhibited the hypoxia-induced mitochondrial fragmentation in NRCMs (Figures 5(h) and 5(i)).

## 4. Discussion

To our knowledge, we demonstrated for the first time that maternal hypoxic exposure during the late stages of fetal development results in decreased anabolism and increased catabolism of PAs in the cardiac tissue of newborn rats. Physiological levels of PAs are critical for cell function. PAs are ROS scavengers, protecting DNA, proteins, and lipids from oxidative damage [48]. More importantly, PAs exert positive effects during embryonic and fetal development, implantation, embryonic diapause, placentation, and angiogenesis [49]. Evidence shows that ODC activity is rapidly increased in the rat uterus after embryo implantation, which brings about the accumulation of PAs in the uterus, placenta, and fetus [50]. In our present study, ODC expression decreased while SSAT expression increased in the cardiac tissues of newborn rats exposed to IUH, which led to reduced levels of protective PAs that was coupled with an increase in the by-product H_2_O_2_ and production of 3-aminoacetaldehyde due to PA catabolism, which was sufficient to cause oxidative damage.

Experimental evidence from human and animal models indicates that IUH-induced placental insufficiency exposes the developing fetus to oxidative and hemodynamic stress, which in turn directly and indirectly impacts the developing cardiovascular system [51]. Thus, we speculated that metabolic disturbance in PAs leads to decreased PAs in the cardiac tissue of newborn offspring, which can be traced back to the decrease in PA levels in placental tissue and the fetus during fetal development and maternal hypoxia. Several studies support this notion; for example, Basystyi [52] revealed that PA concentrations in erythrocytes in the blood of pregnant women with IUGR are drastically lower than those of pregnant women with a normal physiological pregnancy and that the progression in PA imbalance depended on the severity of fetal growth retardation in pregnant women [52]. Another study reported that maternal blood PAs are possibly of fetal origin and that a modified pattern in maternal PAs may indicate insufficient fetal growth [53]. A recent study also suggested that placental PA metabolism differs depending on fetal growth restriction and that maternal serum levels of PA metabolites are strongly associated with obstetrical syndromes [54]. Nonetheless, more evidence is required to explore the role of PAs in the interactions between the fetus and mothers and in fetal and neonatal heart development.

In the present study, we also found that maternal hypoxia exposure led the newborn offspring to exhibit significantly decreased BW and HW and elevated apoptosis and fibrosis and caused cardiomyocytes to exit from the cell cycle ahead of schedule, as indicated by an increase in the number of binucleated cardiomyocytes and MCM2-positive nuclei. This indicates that the IUH resulted in impaired postnatal cardiac growth plasticity. Similarly, several studies also demonstrated programming effects of prenatal hypoxia in the mammalian hearts of IUH rodent model offspring [43, 55, 56]. It is important to mention that a number of studies reported that maternal dietary supplementation with Arg, a major substrate of PA production, increases BW and decreases perinatal mortality in rat, ewe, and rabbit models exposed to IUGR during the gestation period [22, 57, 58]. Moreover, supplementation with Arg was sufficient to increase SPD concentrations in colonic tissue and blood [59, 60]. Of note, a previous study revealed that SPD, but not SP, is essential for early development, organogenesis, and color pattern formation and that maternal supplementation of SPD synthase in zebrafish is sufficient to rescue early developmental defects [34]. A similar earlier study also suggested that uterine and fetal SPD concentrations show sustained elevation during the early stage of gestation in mice, whereas the SP concentration remains relatively stable. Furthermore, they demonstrated the essential role of SPD in murine embryogenesis using the highly selective ODC inhibitor DFMO, which markedly reduced ODC activity and PU and SPD concentrations in uterine tissue. Administration of DFMO results in fetal developmental failure between days 9 and 12 of gestation. Mice, rat, and rabbit were treated with DFMO during the late stage of gestation showing a significant difference from controls in average fetal numbers and weight [61, 62]. DFMO results in depletion of Pu and SPD; however, only incomplete depletion of SP occurs [63].

The broad role of SPD in disease and health has recently received increased attention [31, 32]. Therefore, we evaluated the effect of PA SPD administration during late pregnancy stages on the newborn myocardium by employing a rat model of prenatal hypoxia. Our findings demonstrated that SPD supplementation to the hypoxic mother rescued neonatal BW and HW, decreased the ratio of HW to BW, and maintained good myocardial histological structures in newborn rats. SPD treatment also decreased the ratio of BAX to BCL2 expression and percentage of TUNEL-positive nuclei as well as inhibited myocardial fibrosis (collagen deposition) within the myocardium of IUH-exposed neonates, reversing the effects seen under hypoxia exposure. Conversely, DFMO given to the mother during the late stage of gestation abolished the SPD-mediated protection for the neonatal heart, and this may be due to the deep depletion of cardiac polyamine pool especially SPD by DFMO on one hand; on the other hand, DFMO given to the mother suffering from IUH could cause more serious damage to the placenta and developing fetus than IUH treatment, both of which together contributed to the failure of the protection induced by exogenous SPD. These results indicate that SPD treatment in utero is beneficial for preventing newborn cardiac impairment induced by maternal hypoxia.

Mitochondria are a major source of ROS during prenatal hypoxia, which leads to placental and neonatal oxidative stress and fetal programming of CVD [5]. Neary et al. [47] reported that growth restriction following uteroplacental insufficiency programs reduced cardiac gene expression of the key mitochondrial antioxidant enzyme MnSOD in offspring in later postnatal life [47]. A recent study also revealed that prenatal hypoxia reduced the expression of subunits of the electron transport chain mitochondrial proteins, thereby reducing the mitochondrial respiration rate in the offspring hearts [9]. In addition, other studies showed that maternal exposure to adverse factors (placental insufficiency or high altitudes) during pregnancy will program fetal mitochondria [64]. Similar to these studies, our findings demonstrated that the hearts of newborn offspring exposed to maternal hypoxia showed a reduced mitochondrial area and proportion of mitochondria within cardiomyocytes, decreased mitochondrial respiratory function, and lower antioxidant SOD levels. These results indicate that fetal oxidative stress and mitochondrial dysfunction induced by IUH may persist until after birth, leading to cardiac impairment in the newborn offspring. As mitochondria provide energy for proper cell function, a reduction in number or functional alterations are likely to be detrimental for cells, particularly for cardiomyocytes with high energy requirements [65]; thus, maternal hypoxia is especially detrimental for the developing neonatal heart.

Previous studies have shown that prenatal antioxidant supplementation with resveratrol [66], ascorbic acid [67], or melatonin [68] as well as physical exercise [69] can rescue cardiovascular dysfunction in the fetus during hypoxia and prevent the programming of CVD in adults. In the present study, SPD treatment during hypoxic pregnancies promoted morphological and respiratory function recovery of myocardium mitochondria and elevated SOD levels in the hearts of newborn offspring compared with those exposed to prenatal hypoxic conditions. SPD almost completely blocked the increase in mitochondrial ROS levels and membrane potential depolarization in NRCMs under hypoxic conditions. Conversely, DFMO treatment abolished the SPD-induced effects in vivo and in vitro. Thus, our findings provide strong evidence that maternal SPD treatment can protect the fetus and newborns from hypoxia-induced cardiac oxidative stress and mitochondria injury during hypoxia-complicated pregnancies.

Cardiac mitochondria are dynamically regulated to generate and consume enormous amounts of ATP to support the constant pumping action of the heart. Excessive mitochondrial fission was shown to inhibit mitochondrial respiratory function and increase ROS production [70], whereas the inhibition of mitochondrial fusion can result in loss of mitochondrial DNA [71]. Our present study showed that prenatal hypoxia led to a decrease in mitochondrial biogenesis and an imbalance in mitochondrial dynamics, which was reversed by prenatal SPD treatment. After birth, the heart commences its aerobic metabolism and cardiac mitochondrial biogenesis is increase [72]. Mitochondrial biogenesis regulates mitochondrial dynamics via effects on fusion and fission for mitochondrial maturation in postnatal hearts [73, 74]. Recent evidence from animal experiment implicates that MFN1, MFN2, and Drp1 are essential for postnatal heart development and metabolic remodeling in human beings and mice [71, 75]. Our findings support the notion that SPD can regulate the IUH-induced imbalance in mitochondrial dynamics by inhibiting fission and promoting fusion, which improves mitochondrial function and alleviating myocardial injury in neonatal rats.

There are some limitations to our study, however. For example, hypoxia exposure during pregnancy often decreases maternal food intake, which results in maternal undernutrition. Reduced nutrient intake during pregnancy is associated with the development of a “thrifty phenotype” in offspring, which leads to an increased prevalence of CVD in adulthood. In the present study, maternal food intake was not quantified; thus, we cannot exclude the effects of maternal undernutrition on the pathophysiological changes seen in newborn hearts. Moreover, we established an animal model of IUH by exposing pregnant rats to hypoxia for 4 h per day. Indeed, this is a model of chronic intermittent hypoxia (CIH) rather than a chronic hypoxia pregnancy. In clinical studies, CIH was found to be an important pathophysiological change induced by obstructive sleep apnea-hypopnea syndrome (OSAHS). Evidence shows that OSAHS affects up to one-third of women by the third trimester [76]. OSAHS leads to the upregulation of oxidative stress and pathways of inflammation [77], which are also mechanisms of chronic hypoxia. Finally, it should be mentioned that the PAs include SP, SPD, and their precursor diamine PU. Both SP and SPD can act as direct ROS scavengers, whereas PU exhibits low efficacy in ROS neutralization [63]. In the present study, we showed that maternal SPD supplementation ameliorates mitochondrial function via inhibition of mitochondrial oxidative stress in the hearts of newborns exposed to IUH, but we did not compare the effects of SP, SPD, and PU. In the future, we aim to compare the cardioprotective effects of these PAs on newborn rats exposed to IUH.

## 5. Conclusions

The present study demonstrated that prenatal exposure to hypoxia leads to decreased synthesis and increased breakdown of cardiac PA in neonatal rats. Exogenous SPD administration to hypoxic mothers during pregnancy showed beneficial effects on cardiac fibrosis, cell proliferation and apoptosis, cardiac mitochondrial ROS levels, and mitochondrial fission/fusion balance in the neonatal rats. Our study findings may also aid the development of preventative or therapeutic strategies aimed at IUH offspring to prevent adult CVD. However, the effects of prenatal SPD treatment on fetal cardiac development and the pathways that link abnormal changes during fetal development with those in the offspring still need to be addressed in future studies.

## Data Availability

The data used to support the findings of this study are available from the corresponding authors upon request.

## Supplementary Materials

Supplementary Materials The detailed statistical analysis of the comparison between the Hpx-Spd group and the control group, the Hpx-Spd-DFMO group and the control group, and the Hpx-Spd-DFMO group and the Hpx group of all observation items of the newborn rat heart was provided.
