# Corrigendum to “Spermidine Prevents Heart Injury in Neonatal Rats Exposed to Intrauterine Hypoxia by Inhibiting Oxidative Stress and Mitochondrial Fragmentation”

**DOI:** 10.1155/2019/8695089

**Published:** 2019-09-04

**Authors:** Nannan Chai, Hao Zhang, Lingxu Li, Xue Yu, Yan Liu, Yan Lin, Lina Wang, Jiamin Yan, Sazonova Elena Nikolaevna, Yajun Zhao

**Affiliations:** ^1^Department of Pathophysiology, Harbin Medical University, Harbin 150086, China; ^2^Department of Biochemistry, Harbin Medical University, Harbin 150086, China; ^3^Department of Pathophysiology, Qiqihar Medical University, Qiqihar, Heilongjiang 161006, China; ^4^Laboratory Center of Molecular Biology, Harbin Medical University, Harbin 150086, China; ^5^Department of Physiology, Far Eastern State Medical University, 680000, Russia; ^6^Department of Nursing, Medical School of Chifeng University, Chifeng 024000, China; ^7^Pathology Department, First Affiliated Hospital of Soochow University, Suzhou 215006, China

In the article titled “Spermidine Prevents Heart Injury in Neonatal Rats Exposed to Intrauterine Hypoxia by Inhibiting Oxidative Stress and Mitochondrial Fragmentation” [1], the authors made a typographical error in Figure 5(d) during the proofreading stage as an extra PGC-1*α* was added in the section of the protein name. However, it should be removed. The authors apologize for this error and confirm that it does not affect the conclusions or the results of the article.

The corrected version of Figure 5 is as follows.

## Figures and Tables

**Figure 1:**
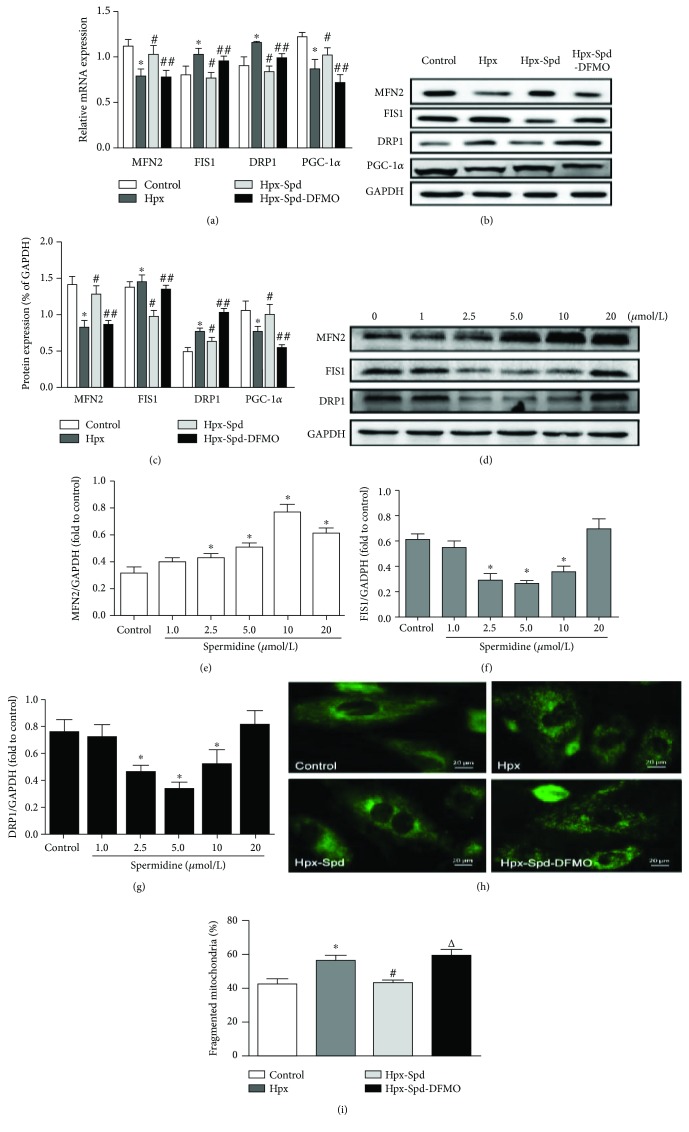
Effects of SPD on mitochondrial biogenesis and fission/fusion dynamics in the offspring myocardium and NRCMs exposed to hypoxia. (a) mRNA expression levels of myocardial MFN2, FIS1, DRP1, and PGC-1*α* were determined by qRT-PCR; GAPDH was used as an internal control (*n* = 6). (b) Protein levels of MFN2, FIS1, DRP1, and PGC-1*α* in the offspring myocardium collected from the control, Hpx, Hpx-Spd, and Hpx-Spd-DFMO groups were assessed by western blotting. (c) Quantification of MFN2, FIS1, DRP1, and PGC-1*α* protein levels normalized to GAPDH in each group (*n* = 4). Data are shown as the mean ± SEM. ^∗^*P* < 0.05 versus the control, ^#^*P* < 0.05 versus the Hpx group, and ^△^*P* < 0.05 versus the Hpx-Spd group. (d) Western blotting showed a dose-dependent effect of SPD (0, 2.5, 5, 10, and 20 *μ*mol) on the protein levels of MFN2, FIS1, and DRP1 in NRCMs after exposure to hypoxia. Quantitative analysis of (e) MFN2, (f) FIS1, and (g) DRP1 protein levels normalized to that of GAPDH. Data are shown as the mean ± SEM. ^∗^*P* < 0.05 versus the control group (0 *μ*mol/L SPD treatment; *n* = 6). (h) Detection of fused and fragmented mitochondria in NRCMs by MitoTracker Green staining (magnification: 400x). (i) The percentage of fragmented mitochondria. *n* = 6 for each group. Data are shown as the mean ± SEM. ^∗^*P* < 0.05 versus the control group, ^#^*P* < 0.05 versus the Hpx group, and ^△^*P* < 0.05 versus the Hpx-Spd group.
