# Supplementary material for: Spermidine Prevents Heart Injury in Neonatal Rats Exposed to Intrauterine Hypoxia by Inhibiting Oxidative Stress and Mitochondrial Fragmentation
Source: Oxid Med Cell Longev. 2019 May 14;2019:5406468. doi: 10.1155/2019/5406468 (PMC6537013; doi:10.1155/2019/5406468)
Supplement: Supplementary Materials — The detailed statistical analysis of the comparison between the Hpx-Spd group and the control group, the Hpx-Spd-DFMO group and the control group, and the Hpx-Spd-DFMO group and the Hpx group of all observation items of the newborn rat heart was provided. [file 5406468.f1.pdf]

The statistical analysis of the difference between Hpx-Spd group and Control group, Hpx-Spd-DFMO group and Control, and Hpx-Spd-DFMO group and Hpx group.

1. Statistical analysis of the changes of body weight

Hpx-Spd *vs.* Con, $P < 0.05$

Hpx-Spd-DFMO *vs.* Con $P < 0.05$

Hpx-Spd-DFMO *vs.* Hpx $P > 0.05$

2. Statistical analysis of changes of heart weight

Hpx-Spd *vs.* Con, $P < 0.05$

Hpx-Spd-DFMO *vs.* Con, $P < 0.05$

Hpx-Spd-DFMO *vs.* Hpx, $P > 0.05$

3. Statistical analysis of changes of HW/BW

Hpx-Spd *vs.* Con, $P < 0.05$

Hpx-Spd-DFMO *vs.* Con, $P < 0.05$

Hpx-Spd-DFMO *vs.* Hpx, $P > 0.05$

4. Statistical analysis of the changes of binucleated cardiomyocytes

Hpx-Spd *vs.* Con $P > 0.05$

Hpx-Spd-DFMO *vs.* Con $P < 0.05$

Hpx-Spd-DFMO *vs.* Hpx $P>0.05$

5. Statistical analysis of the changes of the percentage of MCM2-positive cells

Hpx-Spd *vs.* Con, $P > 0.05$

Hpx-Spd-DFMO vs. Con, $P < 0.05$

Hpx-Spd-DFMO vs. Hpx, $P>0.05$

6. Statistical analysis of the changes of the percentage of TUNEL-positive nuclei

Hpx-Spd vs. Con, $P > 0.05$

Hpx-Spd-DFMO vs. Con, $P < 0.05$

Hpx-Spd-DFMO vs. Hpx, $P>0.05$

7. Statistical analysis of the changes of the expression of BAX and BCL2 proteins

Hpx-Spd vs. Con, $P > 0.05$

Hpx-Spd-DFMO vs. Con, $P < 0.05$

Hpx-Spd-DFMO vs. Hpx, $P<0.05$

8. Statistical analysis of the changes of interstitial fibrotic areas in ventricle sections

Con versus Hpx-Spd vs. Con, $P > 0.05$

Hpx-Spd-DFMO vs. Con, $P < 0.05$

Hpx-Spd-DFMO vs. Hpx, $P>0.05$

9. Statistical analysis of the quantification of the area of cells occupied by mitochondria (%)

Hpx-Spd vs. Con, $P > 0.05$

Hpx-Spd-DFMO vs. Con, $P < 0.05$

Hpx-Spd-DFMO vs. Hpx, $P>0.05$

10. Statistical analysis of the changes of the quantitative mitochondrial area

Hpx-Spd vs. Con, $P > 0.05$

Hpx-Spd-DFMO vs. Con, $P < 0.05$

Hpx-Spd-DFMO vs. Hpx, $P>0.05$

11. Statistical analysis of the changes of mitochondrial state 3 respiration

Hpx-Spd vs. Con, $P > 0.05$

Hpx-Spd-DFMO vs. Con, $P > 0.05$

Hpx-Spd-DFMO vs. Hpx, $P>0.05$

12. Statistical analysis of the changes of mitochondrial state 4 respiration

Hpx-Spd vs. Con, $P > 0.05$

Hpx-Spd-DFMO vs. Con, $P > 0.05$

Hpx-Spd-DFMO vs. Hpx, $P>0.05$

13. Statistical analysis of the changes of the respiratory control rate (RCR)

Hpx-Spd vs. Con, $P > 0.05$

Hpx-Spd-DFMO vs. Con, $P > 0.05$

Hpx-Spd-DFMO vs. Hpx, $P>0.05$

14. Statistical analysis of the changes of SOD proteins expression

Con versus Hpx-Spd, $P > 0.05$

Con versus Hpx-Spd-DFMO, $P < 0.05$

Hpx versus Hpx-Spd-DFMO, $P>0.05$

15 Statistical analysis of the expression changes of Mfn2, Fis1, Drp1 and PGC-1α protein in cardiac tissue

Hpx-Spd vs. Con:

Mfn2, $P > 0.05;$ Fis1, $P > 0.05;$ Drp1, $P > 0.05;$ PGC-1α, $P > 0.05$

Hpx-Spd-DFMO vs. Con：

Mfn2, $P<0.01$； Fis1, $P > 0.05$； Drp1, $P > 0.05$； PGC-1α, $P < 0.001$

Hpx-Spd-DFMO vs. Hpx:

Mfn2, $P > 0.05$； Fis1, $P > 0.05$； Drp1, $P > 0.05$； PGC-1α, $P > 0.05$
